# Multidermatomal herpes zoster involving CN V3 and C2 territories with simultaneous vestibulocochlear deficit: A case report

**DOI:** 10.1097/MD9.0000000000000284

**Published:** 2023-07-25

**Authors:** Taesik Jung, ChanEui Hong, Jung Eun Shin, Chang-Hee Kim

**Affiliations:** a Department of Otorhinolaryngology-Head and Neck Surgery, Konkuk University Medical Center, Research Institute of Medical Science, Konkuk University School of Medicine, Seoul, Republic of Korea.

**Keywords:** cervical nerve, hearing loss, trigeminal nerve, varicella zoster virus, vertigo

## Abstract

Herpes zoster is characterized by the presence of unilateral, localized vesicular rash in a dermatomal distribution. Although in most cases herpes zoster affects a single dermatome, multidermatomal involvement may occur in rare cases.

**Patient concerns:**

In this case report, we describe an immunocompetent patient with multidermatomal herpes zoster involving the cranial nerve (CN) V3 and C2 territories with simultaneous vestibulocochlear deficits. We demonstrate the results of temporal bone magnetic resonance imaging and discuss the pathophysiological mechanism of the patient’s characteristic clinical manifestation.

**Diagnoses:**

The diagnosis of multidermatomal herpes zoster involving CN V3 and C2 territories with simultaneous vestibulocochlear deficit was made. Vestibular function test and pure tone audiometry revealed ipsilateral vestibular impairment and hearing loss.

**Interventions:**

The patient was admitted for treatment with systemic steroids and antiviral agents.

**Outcomes:**

After treatment, vertigo and hearing loss improved, and the skin lesions healed well without scarrings.

**Lessons:**

Considering that the afferent sensory fibers of the CN V and C2 converge in the trigeminocervical nucleus, herpes zoster involving contiguous dermatomes of CN V3 and C2 in the patient may be explained by neural-to-neural spread. And considering that temporal bone magnetic resonance imaging revealed enhancement of the dura of the internal auditory canal without enhancement of CN VII or VIII, the vestibulocochlear deficits in the patient may be caused by inflammation of the inner ear transmitted via the cerebrospinal fluid.

## 1. Introduction

Varicella zoster virus (VZV), also known as human herpesvirus 3, is a double-stranded DNA virus belonging to the family *Herpesviridae*. VZV infection is species-specific to humans. Herpes zoster infection is caused by reactivation of VZV in the cell bodies of neurons. Initial infection with VZV is called varicella or chickenpox and usually occurs in childhood; after the initial infection, a latent infection is established in the cranial nerve ganglia or sensory dorsal root. Under some conditions, the dormant VZV reactivates and migrates along the nerve axons to the skin, causing vesicular eruptions that show a localized dermatomal distribution. Herpes zoster generally manifests as painful vesicular eruptions along the unilateral distribution of the dermatome, most commonly affecting a single thoracic truncal dermatome. Less commonly, involvement of the cervical dermatome or cranial nerves (CNs), especially CN V and CN VII, has been reported.^[1–3]^ Herpes zoster oticus (HZO) refers to VZV reactivation within CN VII, and is characterized by an erythematous vesicular rash in the external ear and otalgia. When accompanied by ipsilesional facial weakness, a diagnosis of Ramsay Hunt syndrome can be made.^[4]^ In HZO, vestibulocochlear symptoms such as hearing loss, tinnitus, and vertigo are often present, and the pathological mechanism of vestibulocochlear deficit has been suggested to be directional spread of VZV to CN VIII from CN VII or CN VIII via the cerebrospinal fluid.^[5–8]^

Here, we present a 35-year-old woman with herpes zoster in multiple adjacent dermatomes involving CN V3 and C2, concomitant with peripheral-type vertigo and hearing loss on the ipsilateral side.

## 2. Case presentation

**Figure 1.**
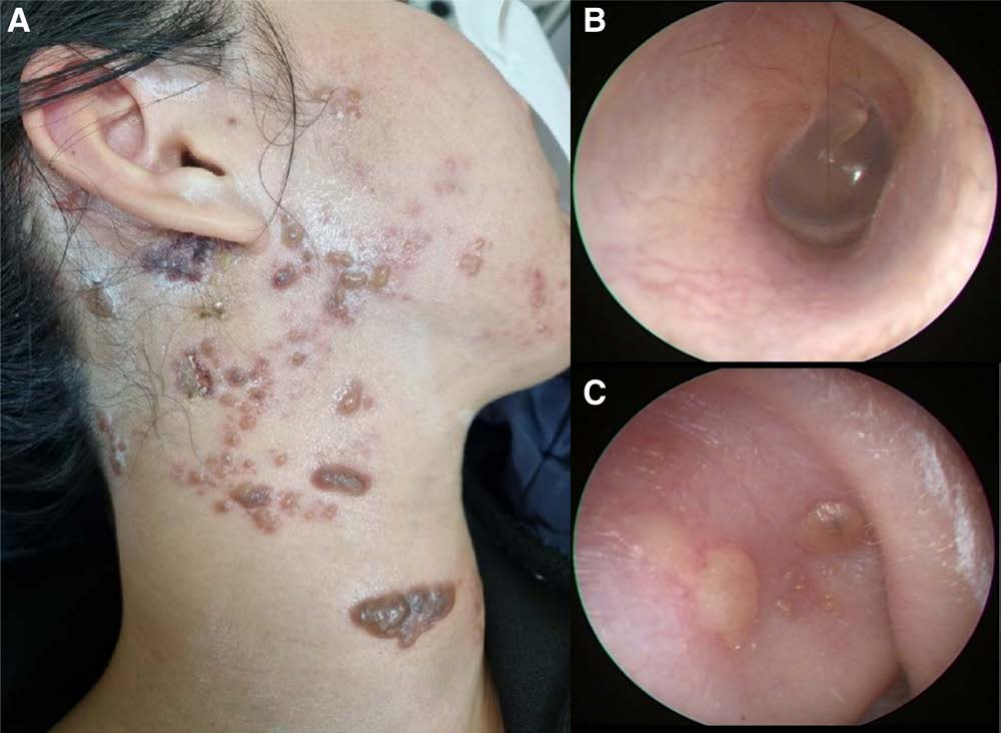
(A) A painful vesicular rash is observed in the distribution of the right C2 and mandibular branches of trigeminal nerve (V3) dermatomes. (B) The right external auditory canal and tympanic membrane are normal in appearance. (C) Vesicles are present at the antihelix and the triangular fossa of the right auricle.

**Figure 2.**
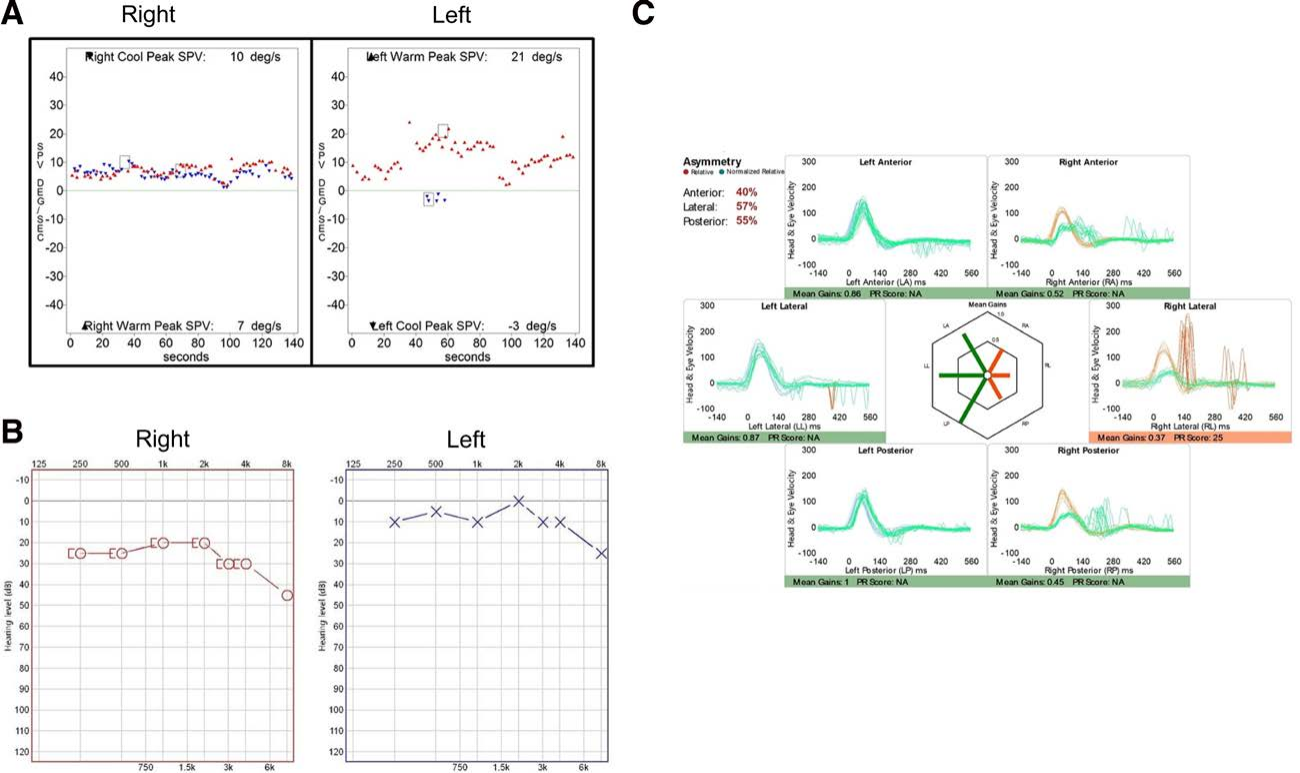
(A) A bithermal caloric test revealed right unilateral weakness (canal paresis of 78% on the right side). (B) Pure tone audiometry showed mild sensorineural hearing loss on the right side. (C) Video head impulse tests revealed decreased vestibulo-ocular reflex gain in the right anterior, lateral, and posterior semicircular canals.

**Figure 3.**
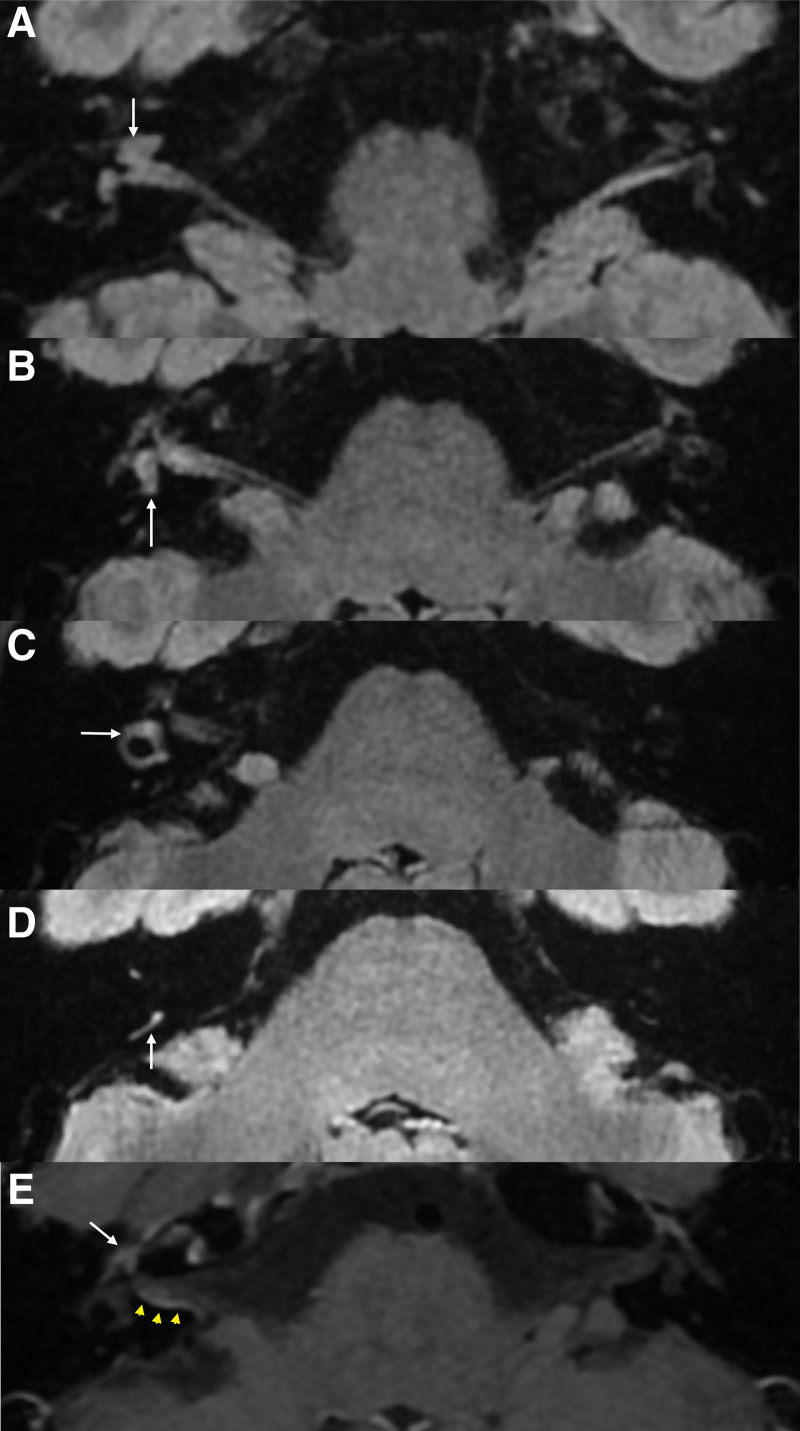
Four-hour-delayed postcontrast FLAIR temporal bone MRI demonstrated enhancement of the right cochlea (A, white arrow), the right vestibule (B, white arrow), the right lateral semicircular canal (C, white arrow), and the right posterior semicircular canal (D, white arrow). (E) The right facial nerve (white arrow) does not show more enhancement than the left facial nerve, and the dura along the posterior wall of the internal auditory canal (yellow arrowheads) is enhanced in postcontrast T1-weighted image. FLAIR = fluid attenuated inversion recovery, MRI = magnetic resonance imaging.

A previously healthy 35-year-old woman visited the emergency room with painful erythematous vesicles, pustules, and scabs of various stages on her right neck, cheek, scalp, and mandibular area (Fig. 1A). The patient reported that the pain first developed in the infraauricular area 7 days prior and that the vesicles appeared in that area 5 days prior. The range of vesicular eruption extended to the scalp and neck, and the patient was prescribed an antiviral agent at the local clinic. However, rotatory vertigo and right-sided hearing loss with tinnitus developed 3 days prior. Otoendoscopic examination revealed normal appearance of the right tympanic membrane and external auditory canal (Fig. 1B) and several vesicles in the skin around the antihelix and triangular fossa of the auricle (Fig. 1C). Thus, a diagnosis of multidermatomal herpes zoster involving the C2 and CN V3 dermatomes was made. Left-beating horizontal-torsional spontaneous nystagmus obeying Alexander’s law was observed on video nystagmography (Video S1, Supplemental Digital Content, http://links.lww.com/MD-CASES/A23). The direction of nystagmus did not change when the head position was changed. No abnormal findings were observed on neurological examinations such as the cerebellar function test and lower cranial nerve examination. Facial weakness was not observed on either side. A bithermal caloric test showed right unilateral weakness with a canal paresis value of 78% (Fig. 2A). Clinical symptoms and signs suggestive of meningeal irritation were negative. Polymerase chain reaction for varicella zoster virus was positive in specimens collected from the patient’s neck vesicles. Pure tone audiometry revealed mild sensorineural hearing loss on the right side with an average threshold of 23.8 dB in the right ear compared to 6.3 dB in the left ear (Fig. 2B). Video head impulse tests elicited reduced vestibulo-ocular reflex gains in all 3 semicircular canals on the right side (Fig. 2C). Four-hour-delay postcontrast 3D fluid attenuated inversion recovery temporal bone magnetic resonance imaging (MRI) showed enhancement of the right cochlea (arrow, Fig. 3A), the vestibule (white arrow, Fig. 3B), the dura along the internal auditory canal (yellow arrowheads, Fig. 3B), the lateral semicircular canal (arrow, Fig. 3C), and the posterior semicircular canal (arrow, Fig. 3D). The facial nerve on the affected side did not show more enhancement than that on the contralateral side (white arrow, Fig. 3E). Enhancement of the dura along the posterior wall of the right internal auditory canal was observed (yellow arrowheads, Fig. 3E).

Following a diagnosis of multidermatomal VZV infection with acute vestibulocochlear deficit, the patient was admitted for treatment with systemic steroids and antiviral agents. The patient also received vestibular suppressants with antiemetics to relieve acute vertigo symptoms and intratympanic steroid injection for acute sensorineural hearing loss. After treatment, vertigo and hearing loss improved, and the skin lesions healed well without scarrings.

This study was approved by our institution’s Institutional Review Board (no. 2023-03-008), and written informed consent was obtained from the patient for publication of this case report details.

## 3. Discussion

This case presentation raises 2 interesting issues that are worth studying. First, herpes zoster involving multiple adjacent dermatomes is very uncommon in young, healthy, immunocompetent patients. Second, except in cases of Ramsay Hunt syndrome, the concomitant vestibulocochlear deficit on the ipsilateral side without evidence of CN VII involvement is also very unusual in individuals with herpes zoster.

Herpes zoster generally develops unilaterally in a single dermatome. The thoracic dermatomes are most commonly affected, followed by the cervical and trigeminal dermatomes.^[9]^ In some patients, herpes zoster extends beyond a single dermatome, and this multidermatomal involvement often occurs in the presence of underlying immunocompromised conditions such as human immunodeficiency virus infection, hematological malignancy and chemotherapy. Multidermatomal herpes zoster involving the trigeminal nerve,^[10–12]^ or cervical^[13]^ or thoracic^[14]^ dermatomes has rarely been reported in immunocompetent patients. The exact mechanism underlying multidermatomal herpes zoster has not been clearly elucidated. Yoon et al^[15]^ reported a case of left Ramsay Hunt syndrome with extensive dissemination of vesicular eruptions to the bilateral scalp, posterior neck, shoulder, upper arm, and posterior upper neck in an immunocompetent elderly patient. The authors assumed that the widespread dissemination of herpes zoster infection may occur through interconnections between ganglia or due to viremia of VZV.^[15]^ Gomez and Chernev reported a 95-year-old patient with disseminated multidermatomal herpes zoster involving the left CN VI-III, the bilateral trunk and bilateral extremities and presented a review of 28 immunocompetent patients with disseminated herpes zoster previously reported in the literature.^[16]^ The results showed that the initial presentation of vesicular rash most frequently occurred in dermatomes innervated by the cranial nerves (42%), followed by the thoracic (27%), lumbosacral (19%), and cervical (15%) dermatomes. Extracutaneous manifestations were reported in twelve cases (43%); among theses, mucosal lesions, central nervous system lesions, cranial or peripheral nerve paresis, Ramsay Hunt syndrome, herpes ophthalmicus, and visceral involvement were observed in 4, 2, 2, 2, 1, and 1 cases, respectively.^[16]^ The authors speculated that widespread dissemination of herpes zoster may be caused by VZV viremia, and this was supported by the previous finding that VZV DNA can be amplified from the serum of immunocompetent patients with localized herpes zoster.^[17]^ Our patient presented with characteristic skin findings of herpes zoster involving contiguous dermatomes of CN V3 and C2. It has been reported that the afferent sensory fibers of the CN V and C2 converge in the trigeminocervical nucleus, and VZV spread between these nerves may occur at this point. Another plausible explanation would be spreading of the virus from skin to skin or from peripheral nerve to peripheral nerve, considering that the dermatomal territories show abundant overlap in head and neck region.^[18]^

Another noteworthy manifestation in our patient was comorbid hearing loss and vertigo due to ipsilateral vestibulocochlear deficit. Because Ramsay Hunt syndrome can be associated with herpes zoster involving CN V or C2, as reported in previous studies,^[15,19,20]^ we suspected herpes zoster oticus as a cause of the vestibulocochlear deficits in this patient. In our previous studies, vestibulocochlear deficits were observed in more than half of patients with Ramsay Hunt syndrome, and postcontrast temporal bone MRI in these patients exhibited enhancement of inner ear structures and of the dura of the internal auditory canal as well as of the facial nerve and the external auditory canal.^[3,5–8,21,22]^ However, our patient did not show facial weakness or enhancement of the facial nerve or the external auditory canal, suggesting that the vestibulocochlear deficits were not accompanied by Ramsay Hunt syndrome or herpes zoster oticus. Concomitant vestibulocochlear deficits and ipsilateral multidermatomal herpes zoster involving the CN V3 and C2 territories in the absence of Ramsay Hunt syndrome has not previously been reported in the literature, and the mechanism of this clinical manifestation is not clear. Although anastomosis between the auriculotemporal branch of CN V3 and the temporofacial division of CN VII has been demonstrated, there is no anastomosis between CN V3 and CN VIII. Thus, in our patient, the possibility of VZV spread via neural anastomoses or interganglionic transmission may be low. When VZV infection extends to the cerebrospinal fluid, central nervous system infections such as encephalitis, encephalomyelitis or meningitis have been reported.^[23–25]^ Eskiizmir et al^[23]^ described an immunocompetent 19-year-old man with Ramsay Hunt syndrome and simultaneous VZV encephalitis. Previously healthy patients with disseminated cutaneous zoster and aseptic meningitis have also been reported.^[24,25]^ Considering that no evidence of meningitis was found in our patient and that temporal bone MRI revealed enhancement of the dura of the internal auditory canal without enhancement of CN VII or VIII, the vestibulocochlear deficits in our patient may be caused by inflammation of the inner ear transmitted via the cerebrospinal fluid.

## 4. Conclusion

To our knowledge, this is the first case report of multidermatomal herpes zoster involving the CN V3 and C2 territories with simultaneous vestibulocochlear deficits. The mechanism of herpes zoster involving contiguous dermatomes of CN V3 and C2 may be neural-to-neural spread, and that of vestibulocochlear deficit may be viral inflammation of the inner ear transmitted via cerebrospinal fluid.

## Author contributions

**Conceptualization:** Chang-Hee Kim.

**Data curation:** Taesik Jung, ChanEui Hong, Jung Eun Shin, Chang-Hee Kim.

**Formal analysis:** Taesik Jung, Jung Eun Shin, Chang-Hee Kim.

**Funding acquisition:** Chang-Hee Kim.

**Investigation:** Taesik Jung, ChanEui Hong, Jung Eun Shin, Chang-Hee Kim.

**Methodology:** Taesik Jung, ChanEui Hong, Chang-Hee Kim.

**Supervision:** Chang-Hee Kim.

**Visualization:** Jung Eun Shin.

**Writing – original draft:** Taesik Jung, Chang-Hee Kim.

**Writing – review & editing:** Chang-Hee Kim.
